# Benefits of Turbid River Plume Habitat for Lake Erie Yellow Perch (*Perca flavescens*) Recruitment Determined by Juvenile to Larval Genotype Assignment

**DOI:** 10.1371/journal.pone.0125234

**Published:** 2015-05-08

**Authors:** Lucia B. Carreon-Martinez, Ryan P. Walter, Timothy B. Johnson, Stuart A. Ludsin, Daniel D. Heath

**Affiliations:** 1 Great Lakes Institute for Environmental Research, University of Windsor, Windsor, ON, N9B 3P4, Canada; 2 Department of Biological Science, California State University, Fullerton, CA, 92831, United States of America; 3 Ontario Ministry of Natural Resources, Glenora Fisheries Station, Picton, ON, K0K 2T0, Canada; 4 Aquatic Ecology Laboratory, Department of Evolution, Ecology, and Organismal Biology, The Ohio State University, Columbus, Ohio, 43212, United States of America; University of South Carolina, UNITED STATES

## Abstract

Nutrient-rich, turbid river plumes that are common to large lakes and coastal marine ecosystems have been hypothesized to benefit survival of fish during early life stages by increasing food availability and (or) reducing vulnerability to visual predators. However, evidence that river plumes truly benefit the recruitment process remains meager for both freshwater and marine fishes. Here, we use genotype assignment between juvenile and larval yellow perch (*Perca flavescens*) from western Lake Erie to estimate and compare recruitment to the age-0 juvenile stage for larvae residing inside the highly turbid, south-shore Maumee River plume versus those occupying the less turbid, more northerly Detroit River plume. Bayesian genotype assignment of a mixed assemblage of juvenile (age-0) yellow perch to putative larval source populations established that recruitment of larvae was higher from the turbid Maumee River plume than for the less turbid Detroit River plume during 2006 and 2007, but not in 2008. Our findings add to the growing evidence that turbid river plumes can indeed enhance survival of fish larvae to recruited life stages, and also demonstrate how novel population genetic analyses of early life stages can contribute to determining critical early life stage processes in the fish recruitment process.

## Introduction

The recruitment process in fishes is complex and influenced by a large number of factors, both biotic and abiotic [1,2]. For many freshwater and marine populations, processes operating during early life stages have been shown to be important determinants of survival to older (and fishable) life stages, with factors that influence food availability and predation risk to larvae being seen as especially important (see reviews by [1,2]). Because nutrient-rich, turbid river plumes that are common to both large lake and coastal marine ecosystems hold great potential to benefit larval fish by enhancing zooplankton availability (via nutrient effects; [3–5]) and reducing risk from visual predators (via turbidity effects; [6–8]), much research has centered on quantifying the role that river plumes play in the fish recruitment process (e.g., [4, 9–12]). Indeed, river inflows and their associated plume fronts have been shown to promote primary and secondary production, as well as foraging during early life stages [13–16]. Despite these generally positive effects of river plumes on fish early life stages, river plumes also can at times inhibit foraging (e.g., [10]D), and evidence that any benefits provided by plumes actually carryover to affect survival at later life stages remains limited (but see [12]).

Documenting whether river plumes provide a survival advantage (or disadvantage) to larvae residing inside the plume versus outside of it requires a means to track relative survival rates between areas. Doing so is difficult, however, in the absence of markers that can identify differential use of the plume during the larval stage. Fortunately, the advent of natural tagging approaches such as otolith microchemistry and molecular genetics has opened the door for addressing this information gap [17]. For example, Reichert et al. [12] used differences in strontium concentrations (Sr) in the otoliths of larval yellow perch (*Perca flavescens*) captured in plume (high Sr) versus non-plume (low Sr) areas to demonstrate better recruitment to the age-0 juvenile stage of larvae residing inside a turbid (Maumee River) plume versus larvae residing outside of this plume in western Lake Erie. While we are unaware of any studies that have used molecular genetics as a natural tag to quantify relative recruitment between individuals residing inside versus outside river plumes during early life stages, its potential use as a tool to track relative recruitment rates would be expected to be high, if genetic divergence could be used to identify river plume habitat use. The genetic divergence could then be used with genotype assignment to determine the relative survival of juvenile fish, similar to approaches used in marine systems to document whether marine protected areas have been affecting the recruitment of both local and geographically distant coral reef fish populations (e.g., [18, 19]). The application of molecular genetic tags for quantifying the subtle mechanisms affecting biological processes in aquatic environments (e.g., habitat use impacts on recruitment; [20, 21]) is based on the underlying effects of dispersal and gene flow on population divergence [22, 23]. Both dispersal and gene flow can be constrained or enhanced by environmental discontinuities such river-plumes [23, 24].

We use microsatellite markers and genotype assignment between life stages (juvenile to larval) to test the hypothesis that survival of larval yellow perch (*Perca flavescens*; YP, hereafter) to the age-0 juvenile stage in western Lake Erie is higher for individuals residing inside a nutrient-rich, turbid river plume than those residing outside of it. Western Lake Erie is a perfect system to test this hypothesis for a number of reasons. First, it is heavily influenced by two rivers that create distinct plumes (water masses) that differ in their chemicophysical characteristics [25, 26] during the spring, a period when the larvae of many economically and ecologically important fishes such as yellow perch are abundant in the ecosystem [2, 27]. Those two plumes constitute the majority of the water mass of the western basin of Lake Erie as the Detroit and Maumee Rivers are by far the dominant tributary rivers. The Maumee River, which discharges into the southern part of the western basin of Lake Erie, has been shown to form a plume that is more turbid, warmer and slightly more biologically productive than the plume formed by the Detroit River, which discharges into the northern portion of the basin [12, 28]. Second, previous research has demonstrated a strong positive correlative relationship between Maumee River plume size and recruitment of western basin YP to the age-0 juvenile stage, a life stage that is very strong predictor of future recruitment to western Lake Erie’s fishery at age-2 [29]. While the mechanisms are not yet known, turbidity’s ability to reduce predation risk (i.e., mitigation of top-down effects) appears to be more important than zooplankton prey supplementation (bottom-up effects) via nutrients [8, 12, 28]. Third, although no previous studies have examined genetic structure in YP larvae in their rearing environment, low levels of genetic structure have been documented in adult YP within the western basin of Lake Erie [30, 31] indicating a high likelihood for larval genetic structure. Given that fish populations can evolve life histories that center on predictable physical phenomena such as river flows and water circulation patterns, and strong selection (predation) gradients appear to exist in Lake Erie that are driven by Maumee River plume formation [8, 12, 28], the possibility that larval YP exhibit genetic structure at the scale of the Detroit and Maumee River plumes exists. Finally, because YP is an ecologically and economically important species in all of the Laurentian Great Lakes that also has a similar life history and level of recruitment variability as many other exploited freshwater and marine fishes [2], we expect our findings and approach to have application to a wide array of ecosystems.

Here we use polymorphic microsatellite markers, population genetic analyses, and genotype assignment to explore the role of river plume-associated turbidity on YP recruitment to the age-0 juvenile stage in the western basin of Lake Erie. We postulate that larval YP will exhibit genetic structure at the scale of the Detroit and Maumee River plumes, and that the genetic structure can be used in conjunction with genotype assignment of juvenile YP to their respective larval cohorts to indirectly estimate first summer relative survival in the two rearing habitats. Towards this end, we 1) tested for spatial genetic structure in larval YP in the western basin of Lake Erie, 2) quantified relative survival to the age-0 juvenile stage (age-0) of larvae residing inside versus outside of the turbid Maumee River plume, and 3) tested for temporal variation in recruitment and population genetic structure. Our analyses identifies survival differences among larval fishes from Western Lake Erie and suggests that river plumes may confer benefits for larval YP survival, adding to a growing understanding of the complex ecology of Lake Erie.

## Materials and Methods

### Fish collections

**Fig 1:**
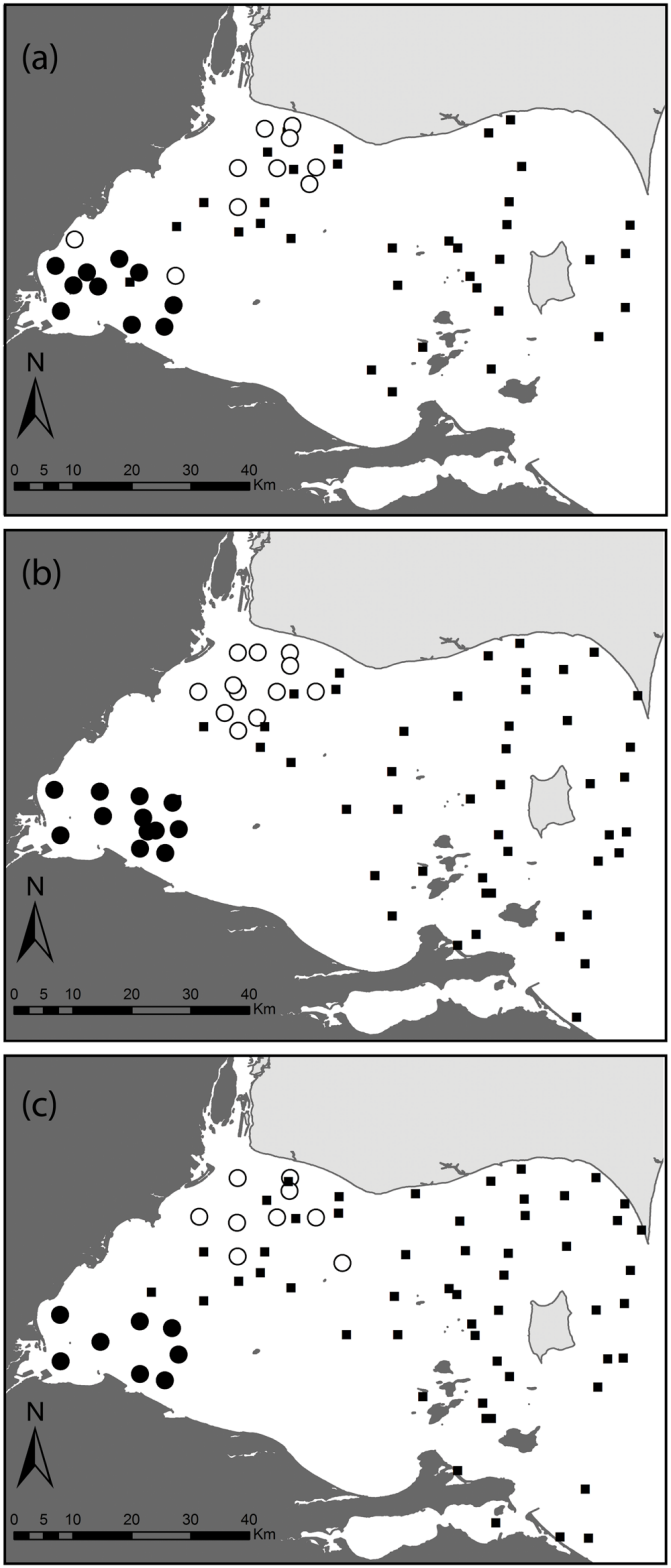
Maps of western Lake Erie with larval (large circles) and juvenile (small black squares) yellow perch sampling sites during spring 2006 (Panel a), 2007 (Panel b) and 2008 (Panel c). Transmissometry values greater than six indicate high turbidity and differentiate the Maumee River plume (dark circles) from Detroit River plume waters (white circles). In 2006, two sites were not used in the analysis due to anomalous turbidity values (eliminated sites are shown as white circles in the Maumee plume; no sample sites were eliminated in 2007 or 2008).

Larval YP were collected weekly at 8 to 12 sites within both northern (Detroit River plume) and southern (Maumee River plume) areas of the western basin of Lake Erie (Fig 1). Moderate-Resolution Imaging Spectoradiometer (MODIS) 250 m resolution, true colour, near real-time imagery from the Terra and Aqua satellites (http://coastwatch.glerl.noaa.gov/) was used to determine the rough boundaries of the Maumee and Detroit River plumes as the turbid waters from the Maumee River are evident in the images (see details in [12]). YP larvae were captured during close to identical periods in the two plumes, with the exception of 2007 when the date of first capture was a week earlier in the Maumee (day 122 versus day 128) and in 2008 when the date of last capture was a month later in the Maumee (day 175 versus day 144).

Fishes collected in Ontario waters of Lake Erie were taken as part of routine fishery monitoring activities conducted by the Lake Erie Management Unit of the Ontario Ministry of Natural Resources and Forestry under License No. 1045675. Fishes collected from Ohio waters were taken under Ohio Division of Wildlife Wild Animal Permit 09–95. Fishes collected from Michigan waters were taken under a Scientific Collector’s Permit-Fish issued by the State of Michigan Department of Natural Resources. No specific permission other than permissions granted through the above permits was required for these locations/fish collections. Collections did not involve endangered or protected species. Larvae were collected using oblique (~1 m from bottom to surface) tows with paired 1 m diameter, metered bongo nets (500 μm and 1000 μm meshes) on a weekly basis from late April through June in 2006, 2007, and 2008. Larvae were humanely sacrificed using ethanol or rapid chilling following the AMVA Guidelines (https://www.avma.org); larvae were preserved in 100% ethanol until identified to species in the laboratory [32]. Fish collection methods were approved by University of Windsor’s Animal Care Committee (ACC) and the Fisheries Management Unit of the State of Michigan Department of Natural Resources prior to collections. Sampling procedures were specifically approved as part of obtaining field permits for all locations.

At each collection site, turbidity was indirectly estimated using a 5 cm path transmissometer (SeaBird SBE19): transmissometry values reflect loss of light transmission with increased water turbidity [12]. Although our sampling site positions were based on satellite images, the real time spatial location of the Maumee River plume was highly dynamic. We thus used weekly transmissometry measurements (as a proxy for turbidity) to corroborate sampling sites assignment to one of the two larval rearing (plume) habitats, hence providing objective criteria for delineating two distinct larval habitats for the genetic analyses.

Age-0 juveniles (i.e., surviving larval YP from the spring of the same year) were collected throughout western Lake Erie in late August of each year via bottom trawl (10.7-m headrope; 13-mm cod-end liner; 3 km/hr tow-speed). These juveniles were collected from 36, 50, and 48 sites within the western basin during 2006, 2007, and 2008, respectively (Fig 1), as part of the annual fisheries-independent assessment surveys conducted by the Ontario Ministry of Natural Resources and the Ohio Department of Natural Resources-Division of Wildlife [29]. All individuals were humanely euthanized and kept frozen until further laboratory analysis. Because juvenile abundance is a strong predictor of future recruitment to the fishery in western Lake Erie [29], juveniles used for this study in each year were subsampled from the catch at each site in proportion to the total catch per unit effort at that site.

### DNA extraction and genotyping

DNA was recovered from fin tissue samples following the plate-based extraction method of [33]. Extracted DNA samples were eluted in 50–100 μL of Tris—EDTA buffer (10 mM Tris, 1.0 mM EDTA, pH 8.0).

Each fish was genotyped at a total of 12 microsatellite loci ([34]; Table 1). PCR amplification was performed in 25 μL reactions with the following components: 1.5 μL of template DNA, 2.5 μL 10x PCR buffer, 2.5 μL of MgCl_2_ (25 mM), 0.3 μL of dNTPs (50 μM of each), 0.2 μL (0.5 μM) of dye labeled primer, 0.2 μL (0.5 μM) of the reverse primer, and 0.10 U Taq polymerase (Applied Biosystems, Foster City, CA). PCR conditions were: initial denaturation at 94°C for 2 min, followed by 35 to 40 cycles of denaturing at 94°C for 15 s, annealing at various temperatures (according to Li *et al*. [34]) for 30 s, extension at 72°C for 30 s, and a final extension of 72°C for 10 min. Microsatellite allele sizes were determined using a LI-COR 4300 DNA analyzer (Lincoln, NE) and scored using GeneImage IR 4.05 (Scanalytics, Inc., Rockville, MD). Microsatellite data are freely available at: http://dx.doi.org/10.5061/dryad.vf0t2

**Table 1:** Number of alleles (N_A_), and observed (H_O_) and expected (H_E_) heterozygosity for the 12 microsatellite loci (Li et al. 2007) used to genotype larval yellow perch (YP) collected in the western basin of Lake Erie during 2006 (06), 2007 (07), and 2008 (08).

		Locus											
Groups		YP85	YP78	YP41	YP109	YP55	YP110	YP96	YP60	YP65	YP49	YP81	YP99
D06	N	121	**127**	126	109	111	116	**119**	**125**	132	127	**118**	111
	N_A_	15	**13**	7	22	9	9	**9**	**6**	10	9	**6**	12
	H_O_	080	**087**	068	082	053	015	**066**	**056**	061	078	**064**	087
	H_E_	084	**085**	060	092	047	015	**049**	**046**	056	064	**051**	085
M06	N	64	62	71	47	66	62	69	64	64	68	61	60
	N_A_	15	11	8	20	5	4	6	9	10	6	14	10
	H_O_	058	077	073	079	061	010	059	069	064	072	054	075
	H_E_	079	083	061	093	055	015	051	058	062	059	075	084
D07	N	199	206	198	204	**206**	191	**208**	**199**	**193**	**202**	200	201
	N_A_	19	12	7	24	**6**	7	**9**	**8**	**8**	**9**	6	13
	H_O_	070	090	065	079	**058**	012	**075**	**070**	**084**	**098**	064	086
	H_E_	073	084	060	093	**051**	013	**057**	**054**	**062**	**067**	061	086
M07	N	53	68	70	54	69	66	**69**	**57**	**66**	59	64	58
	N_A_	17	12	5	20	5	7	**5**	**6**	**7**	6	4	11
	H_O_	091	099	061	096	068	009	**071**	**082**	**079**	088	061	091
	H_E_	086	084	057	094	055	012	**050**	**058**	**057**	065	053	083
D08	N	111	**114**	115	100	**123**	124	113	117	114	119	106	98
	N_A_	18	**13**	7	26	**7**	8	6	7	11	8	5	11
	H_O_	060	**099**	065	077	**074**	012	042	034	068	073	051	083
	H_E_	078	**082**	062	094	**058**	012	037	037	059	061	065	082
M08	N	71	**73**	70	66	**73**	72	65	67	**68**	69	73	71
	N_A_	18	**12**	7	24	**7**	5	7	6	**8**	5	5	11
	H_O_	082	**096**	069	086	**075**	014	045	037	**088**	062	059	087
	H_E_	078	**082**	059	092	**062**	018	042	034	**060**	057	066	085

Groups are denoted by river plume (Detroit or Maumee) followed by the collection year.

Note: Data in bold denotes deviations from HWE (following Bonferroni correction).

### Categorizing larval fish groups

Collected larvae were divided into two habitat groups based on two criteria. First, we initially divided larvae based on their geographic (sampling) location (i.e. northern vs. southern part of the western basin; Fig 1). Second, we used transmissometery (as a proxy for turbidity) estimates to select sites belonging to high and low turbidity areas (Fig 1). The larvae associated with highly turbid southern area will be referred to as the Maumee River plume larval group, whereas the larvae associated with low-turbidity northern area of the basin will be referred to as the Detroit River plume larval group.

### Population genetic analysis

Deviation from Hardy—Weinberg equilibrium (HWE) was tested using 20,000 permutations in TFPGA 1.3 [35]. Significance values for HWE were Bonferroni corrected for multiple simultaneous comparisons. In addition, linkage disequilibrium was determined for all pairs of loci in all larval and age-0 juvenile groups using GENEPOP 4.0.7 [36]. Genetic differentiation was quantified by calculating F_ST_ [37] between larval groups (Detroit vs. Maumee River plumes) within a given year (2006, 2007, or 2008) in ARLEQUIN 3.0 [38]. Pairwise Fisher’s exact tests (10,000 dememorizations and 20,000 permutations; [39]) were performed between larval groups in each year, as well as for temporal variation among sample years for the two larval groups using TFPGA. Analysis of Molecular Variance (AMOVA) was used to partition larval YP genetic variance among years (temporal), between plumes nested within years (spatial), and within plumes, using ARLEQUIN. GENELAND 3.2.4 [40] was used to characterize and visualize spatial genetic relationships among larvae for each sampling year. Ten independent Markov Chain Monte Carlo (MCMC) runs at K = 2 (based on two plumes) were performed using the correlated frequencies model, 10^6^ iterations, and a 2 × 10^5^ iteration burn-in.

#### Genetic assignment of juveniles

Relative contributions of larvae from the two plumes to the mixed population of unknown-origin juveniles was estimated by genetic assignment of the juveniles to their putative larval group (i.e., Maumee or Detroit River plume larval groups), followed by a comparative analysis of relative proportions. For genetic assignment purposes only, both groups of larval fish were genetically screened for first-generation migrants using rank-based self-assignment genotype analysis [41] in GENECLASS 2.0 [42]. The purpose of this analysis was to identify larvae that may have moved between sampling areas, likely due to passive dispersal via water movement, as larval YP are weak swimmers [43]. The probability of genotype assignment to either group (high-turbidity, south-shore Maumee River plume vs. low-turbidity, north-shore Detroit River plume) was estimated for each larva. An individual that showed a < 60% likelihood of self-assignment to its collection group was deemed a migrant and eliminated from subsequent analyses to maximize the probability of successful juvenile assignment to the larval groups. Because our choice of a 60% likelihood threshold was arbitrary, we performed a sensitivity analysis to explore the effect of changing this threshold value on the analysis outcomes. It is important to note that the migrant exclusion was not performed for the population genetic characterization of the two plumes described above, only for the genotype assignment.

Genetic assignment of juveniles to the two river plume groups was independently performed for the three collection years using GENECLASS. Our analyses consisted of a two-step procedure (see Beneteau *et al*. [44]). First, to identify (and eliminate) those individuals that may have come from other (not sampled) larval sources, we performed a Bayesian exclusion assignment [45] with Monte Carlo re-sampling using Paetkau *et al*.’s [46] simulation algorithm (10,000 simulated individuals at an assignment threshold p = 0.05). Based on that Bayesian analysis, we excluded individual juveniles with assignment probabilities < 10% of belonging to either of the two larval source populations (i.e., Detroit or Maumee River larval groups). Second, to assign the remaining individuals to one larval group, we used a rank-based genotype assignment (frequency method; [41]). Successful ranked-based assignments were those with probability ≥ 70% of belonging to one group (hence, the second group assignment probability would be < 30%). Failed assignments (i.e. unknown origin) were those with likelihoods between 30% and 70%. Since the 70% likelihood threshold value was arbitrary, we performed a sensitivity analysis to assess the effect of our choice of threshold value on the outcome of our analysis. Specifically, we varied the threshold from 60 to 90% and estimated the relative number of juveniles assigned to the Detroit versus Maumee plume larval groups.

For juvenile YP successfully assigned to Maumee and Detroit River plume larval groups, genetic differentiation (F_ST_) and pairwise Fisher’s exact tests for population differentiation were conducted in ARLEQUIN and TFPGA, respectively, for 2006, 2007 and 2008 independently. Hierarchical AMOVA was used to partition genetic variance in successfully assigned juveniles among years (temporal), between plumes nested within years (spatial), and within plumes, using ARLEQUIN.

Finally, we investigated whether juveniles that successfully assigned to the Detroit or Maumee River plume larval groups were spatially separated in the western basin of Lake Erie. To do so, we calculated the location of capture (latitude and longitude) for all juveniles and used Students’ t-tests to see if the mean latitude and longitude differed between the Maumee and Detroit River groups.

#### Comparison of plume recruitment

We used information on weekly larval YP abundance differences between the Maumee and Detroit River plumes, as well as results from our successful genotype assignments of juveniles, to quantify differences in recruitment of larvae to the age-0 juvenile stage between the two larval rearing areas. Weekly average abundance of larvae was calculated for each river plume group (total number of larvae / m^3^ averaged over all sites sampled in that week) with analysis of variance (ANOVA) used to quantify temporal stability of weekly larval abundance within each river plume. The highest weekly larval abundance estimate has been shown to provide a good estimate of larval production (Reichert *et al*. 2010); hence, we used peak weekly larval abundance values from both plumes to estimate the YP larval abundance ratio between Detroit and Maumee River plumes. In this study the peak larval YP abundance varied from 40–198 larvae/m^3^.

If recruitment of larvae (survival of larvae to August, when juveniles were sampled) was equal for the Detroit and Maumee River plume habitats, we would expect the Maumee: Detroit ratio of larval abundance to remain constant over time. In turn, we would expect the ratio of assigned juveniles from Maumee versus the Detroit plume areas to be the same as the ratio of larval abundance. We used the larval YP abundance ratio (estimated based on metered bongo net tows; see above) to assess differences in relative recruitment between Detroit and Maumee larvae from the larval to the juvenile stage for each year separately. Expected values for the number of juveniles from each plume in each year were calculated by multiplying the Detroit:Maumee larval abundance ratio by the total number of juveniles successfully assigned for that year. Observed values came from the juvenile genetic assignment results (see above). We compared expected versus observed estimates using Chi-square (χ^2^) tests for each year. If the observed number of juveniles was significantly higher than the expected value for a plume (and would thus be lower than expected for the other plume), then relative recruitment would be higher for that river plume.

## Results

The collection sites for larval fish corresponded well with the water turbidity data in discriminating high transmissometry (> 6.0 m^-1^) Maumee River plume sites from the low transmissometry (< 6.0 m^-1^) Detroit River plume sites. Using a 6.0 m^-1^ transmissometry threshold criterion, only two of 12 sampling sites from the Maumee River plume were removed from our initial spatial classification in 2006, and none were removed from either plume in 2007 or 2008 (Fig 1).

A total of 853 larvae and 403 juveniles were genotyped at 12 microsatellite loci across sampling years (2006–2008), with 5 to 23 alleles per locus. Observed (H_O_) and expected heterozygosities (H_E_) ranged from 0.091 to 0.991 across loci (Table 1). Seventeen of 72 tests revealed significant departures from HWE following Bonferroni correction; however, none was consistent across larval groups or loci (Table 1). In addition, no evidence for linkage disequilibrium was found between any pair of loci after Bonferroni correction.

### Population genetic structure

Genetic differentiation (F_ST_ values) between larvae collected in the Detroit versus Maumee River plumes were 0.0086 (P < 0.001), 0.0054 (P < 0.01) and 0.0082 (P < 0.001) in 2006, 2007 and 2008, respectively (Table 2). Fisher exact tests revealed significant (P < 0.001) differences in allele frequency distributions for all comparisons, both with years (i.e., between Maumee and Detroit larval groups) and among years (i.e., among 2006, 2007 and 2008). AMOVA partitioning showed no significant variance component among years (variance explained = 0.43%, P = 0.22), whereas the between-plume variance component (nested within years) was significant (variance explained = 0.31%, P < 0.001). Within-river plume group variation explained the majority of the variance (variance explained = 99.26%, P < 0.001).

**Table 2:** Microsatellite F_ST_ values (Detroit versus Maumee River plume groups) for both yellow perch larvae and age-0 juveniles collected in western Lake Erie during 2006, 2007, and 2008.

Locus	Larval F_ST_			Juvenile F_ST_		
	2006	2007	2008	2006	2007	2008
YP85	-0.0057	0.0298	0.0323	0.0015	0.0721	0.0403
YP78	-0.0060	-0.0036	0.0043	0.0049	0.0054	0.0020
YP41	-0.0048	-0.0025	0.0076	0.0050	-0.0084	0.0380
YP109	0.0006	-0.0034	-0.0034	0.0298	0.0176	0.0432
YP55	0.0437	-0.0007	0.0162	0.0072	0.0646	0.0259
YP110	-0.0005	-0.0035	0.0016	0.0736	0.0010	0.0331
YP96	-0.0030	-0.0042	0.0007	0.0033	-0.0029	0.0165
YP60	0.0349	-0.0047	0.0058	-0.0041	-0.0042	0.0012
YP65	0.0264	-0.0042	0.0016	0.0058	-0.0035	0.0175
YP49	-0.0016	0.0050	-0.0045	-0.0076	-0.0030	0.0057
YP81	0.0914	0.0010	0.0034	0.0790	0.0894	0.0468
YP99	-0.0014	-0.0008	0.0018	-0.0052	0.0120	0.0018
**All loci**	**0.0086**	**0.0054**	**0.0082**	**0.0143**	**0.0244**	**0.0240**

Values are given for all 12 loci individually, as well as across all loci. The larval F_ST_ estimates are based on all sampled larvae; the juvenile F_ST_ estimates are based on fish assigned to Maumee or Detroit River plumes at a 70% probability threshold.

**Fig 2:**
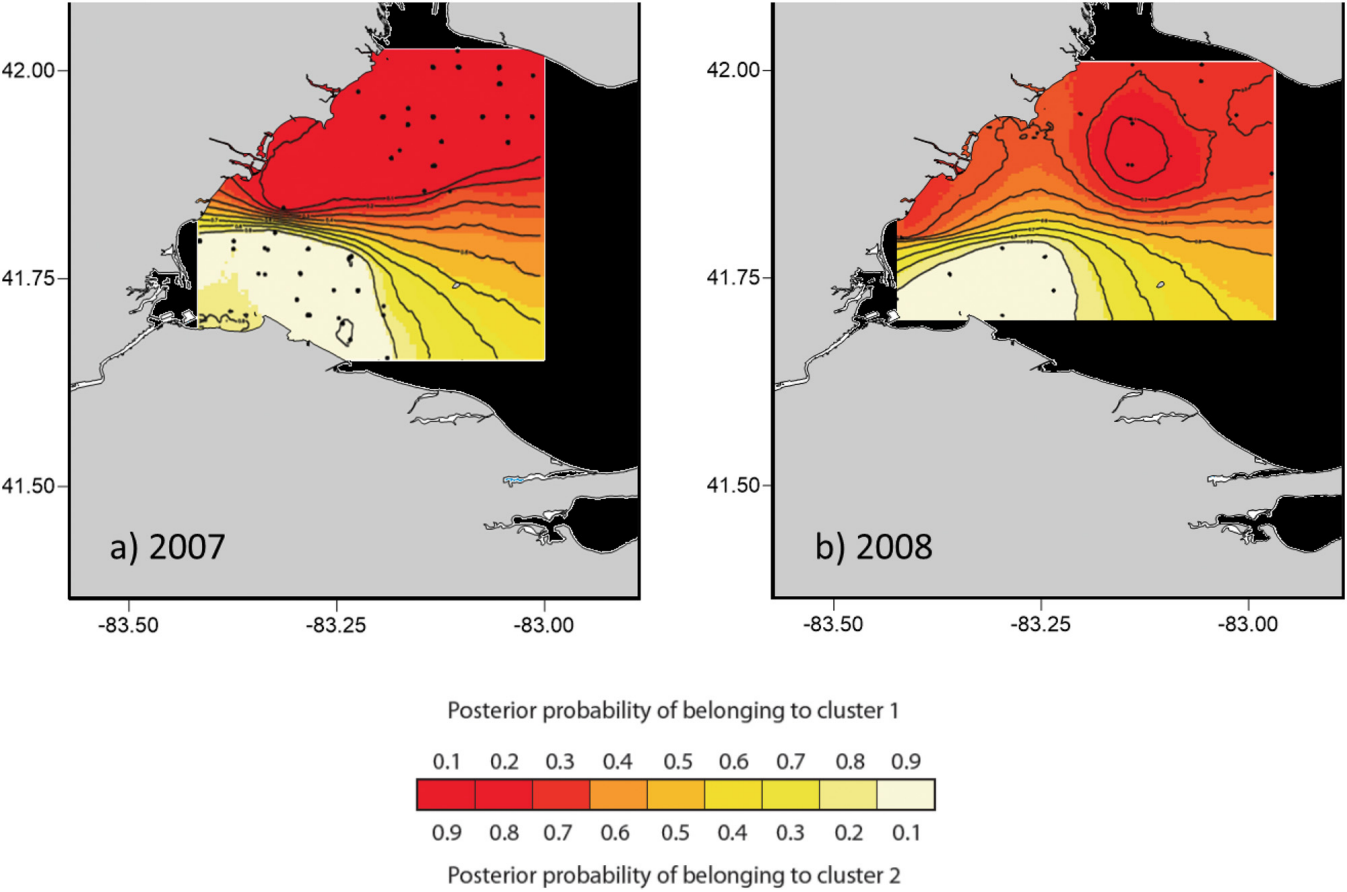
Spatial patterning of western Lake Erie larval yellow perch genetic structure for 2007 and 2008 using GENELAND genetic analysis software. Runs were fixed for two groups (K = 2) for each year. Panels show consensus results of 10 runs for each of two sample years (2007: 10/10; 2008: 6/10). GENELAND failed to converge and no consensus outcome was identified for 2006. Points on the contour maps indicate collection locations of larvae.

Spatial genetic structure corresponding to the two river plumes was identified using GENELAND for 2007 and 2008, but not 2006. Only MCMC runs for samples from 2007 reached convergence as identified by a plateau in the number of iterations versus—ln probability. For 2007, 10 of the 10 independent runs produced identical assignments of individual larvae to two groups corresponding to the Detroit and Maumee River plumes (Fig 2). Datasets for the 2006 and 2008 did not converge with 10^6^ iterations, which is indicative of weak genetic structure within the datasets. For 2008, 6 of 10 runs produced assignments of larvae to two groups consistent with the river plumes (Fig 2). For 2006, all 10 runs produced assignments indicative of population admixture among the plumes (results not shown).

#### Genetic assignment of YOY

The microsatellite genotype assignment analysis for plume membership identified a relatively small number of larvae as likely first-generation migrants: 15% (25 of 160) and 17% (15 of 90) from Detroit and Maumee River plume, respectively, in 2006; 26% (74 of 283) and 13% (11 of 81), respectively, in 2007; and 18% (29 of 153) and 15% (13 of 86), respectively, in 2008. While all larvae were used for the population genetic analyses (above), the putative first-generation migrants were not used in the juvenile assignment analyses.

**Fig 3:**
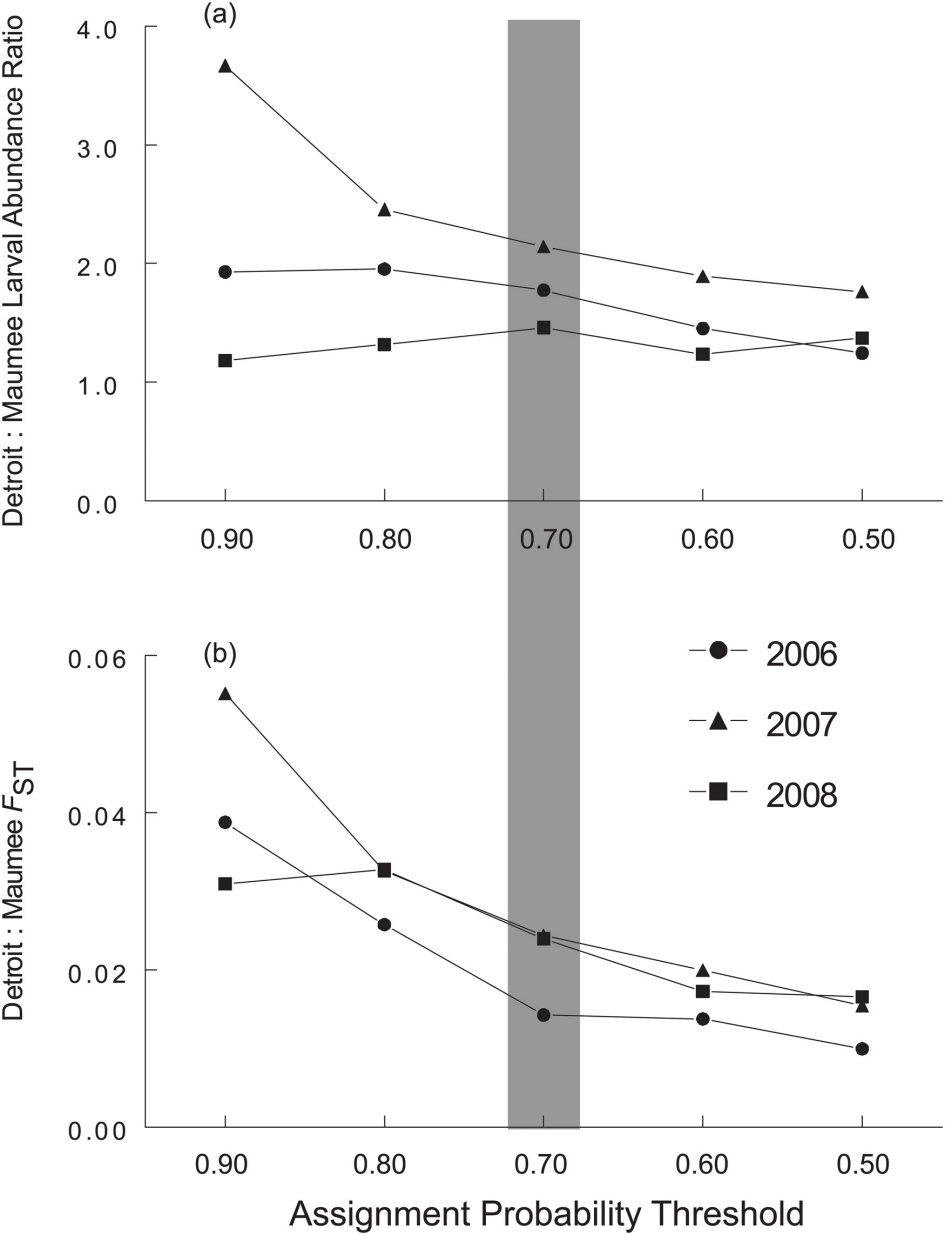
Sensitivity of western Lake Erie yellow perch genotype assignment analyses to changes in assignment likelihood ratio threshold. Panel a) portrays the effect of threshold variation on ratio of age-0 juveniles successfully assigned to the Detroit River versus Maumee River for 2006–2008. Panel b) shows the effect of threshold probability variation on F_ST_ estimates of age-0 juveniles assigned to Maumee River versus Detroit River plume nursery areas for 2006–2008. The shading indicates the threshold used for the analyses.

The percentage of juveniles that were excluded from both possible source populations based on the Bayesian analysis was 0% (0 out of 119), 8% (13 out of 167), and 25% (30 out of 117) in 2006, 2007, and 2008 respectively (Table 3). Although some juveniles were not successfully assigned to either larval rearing area in our rank-based assignment analysis (see “failed assignments” in Table 3), those that did were assigned as Detroit River plume individuals at almost twice the frequency of those assigned to the Maumee River plume in 2006, 2007, and 2008. Further, our sensitivity analysis showed that, although the proportion of juveniles assigned to the Detroit River plume varied with the assignment threshold used, the number of juveniles assigned to the Detroit River plume was always higher than the number assigned to the Maumee River plume (i.e., ratio > 1.0; Fig 3a).

**Table 3:** Genotype assignment of age-0 juvenile yellow perch collected in the western basin of Lake Erie to Detroit versus Maumee River plume larval groups during 2006–2008.

Age-0 Juvenile assignment					
	Detroit	Maumee	Failed	Excluded	Total
2006	55	32	32	0	119
2007	69	33	52	13	167
2008	27	16	44	30	117

Failed assignment indicates that the juvenile genotypes provided an ambiguous likelihood ratio making assignment to either plume uncertain. “Excluded” genotypes are those that had a < 10% probability of belonging to either larval group, likely indicative of an un-sampled source group.

Genetic differentiation (F_ST_) between juveniles successfully assigned to Detroit and Maumee River plumes was 0.011 in 2006, 0.021 in 2007 and 0.029 in 2008, with significant differences in allele frequency distributions in all years (Fisher’s exact test, P < 0.001). To explore the role of our assignment threshold choice on genetic divergence in successfully assigned juveniles, we performed a sensitivity analysis to quantify changes in F_ST_ estimates with varying assignment thresholds. Although lower thresholds did reduce F_ST_ values, the juvenile F_ST_ estimates (Fig 3b) were substantially higher than larval F_ST_ estimates in 2007 and 2008 across all assignment probability thresholds.

All temporal comparisons for juvenile YP allele frequency distribution differences were significant (Fisher’s exact tests, P < 0.001). AMOVA results for juveniles that were successfully assigned to either the Detroit or Maumee River plume showed no variation among years (df = 2, variation = -0.35%, P = 0.68). However, significant variation existed between assigned groups nested within years (df = 3, variation = 1.98%, P < 0.001), with the majority of the variation being explained by within-group effects (df = 458, variation = 98.37%, P < 0.001).

Based on Student’s t- tests, no differences existed between mean latitude and mean longitude of collection sites coordinates for juveniles assigned to the Detroit versus Maumee River plumes in any year (P > 0.05) suggesting that no spatial bias existed in either group (i.e., juveniles from both rearing habitat areas were well-mixed).

### Recruitment differences between plumes

**Fig 4:**
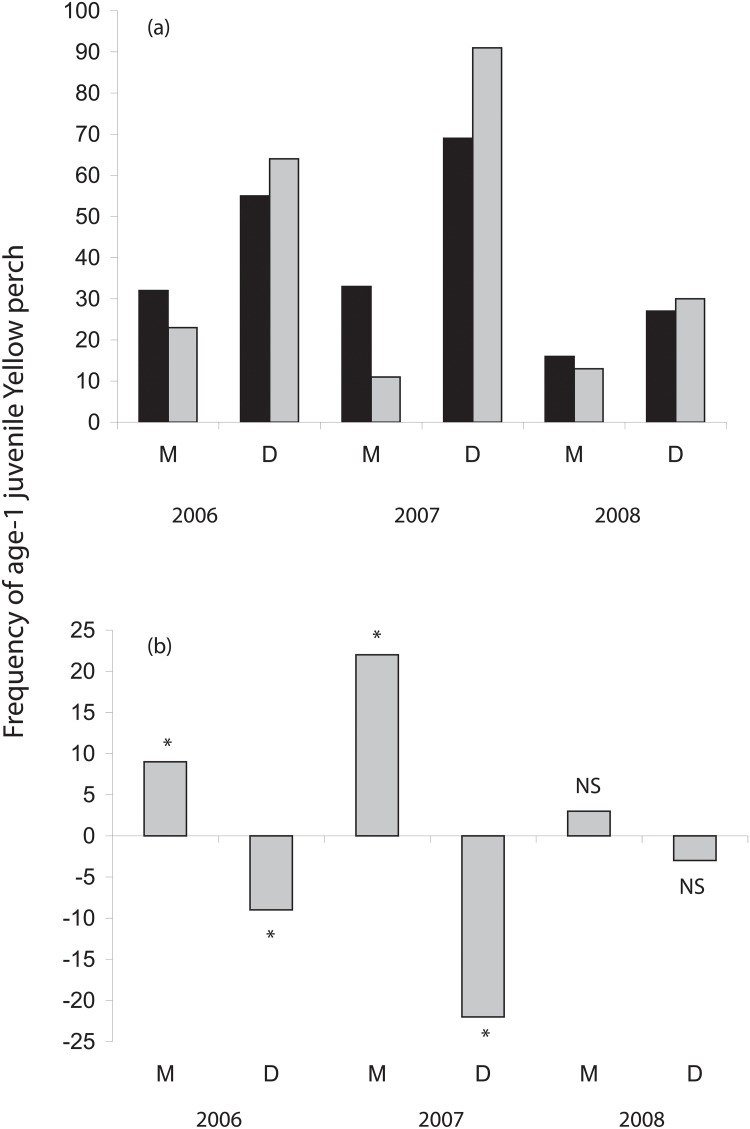
Number of western Lake Erie juvenile yellow perch (YP) assigned to the Detroit (“D”) and Maumee (“M”) River plumes during 2006–2008. Observed (black) and expected (shaded) values in panel a) were calculated based on the larval abundance ratio between Detroit and Maumee River plume groups and genetic assignment results. Residual numbers (observed minus expected) are reported in panel b). Significant differences (P <0.05) between observed and expected values based on Chi-square good of fitness analysis are denoted by asterisks. NS = not significant.

The ratios of peak larval abundance for the Detroit:Maumee River plumes were 73:27 in 2006; 89:11 in 2007, and 70:30 in 2008. No difference in larval YP abundance was found among sample weeks in the Detroit or Maumee River plumes (ANOVA, df = 2, both P > 0.28). Larval YP abundance ratios were used to calculate the *expected* ratio of juveniles from the Detroit River and Maumee River plumes (accounting for the total number of juveniles that were assigned), whereas *observed* values were those obtained using genotype assignment (Table 3). Chi-square analysis revealed significant differences between observed and expected values for 2006 and 2007, but not for 2008 (Fig 4). Specifically, recruitment of larvae to the age-0 juvenile stage was higher than expected in the Maumee River plume and correspondingly lower than expected in the Detroit River plume in 2006 and 2007, whereas we could detect no difference in 2008 (Fig 4).

Finally, we conducted a sensitivity analysis to explore the effect of our choice of a 70% likelihood ratio threshold for juvenile genotype assignment. While we found that the choice of threshold ratio does affect the Detroit:Maumee ratio of juveniles, its effect is minor except at extreme values of the threshold (see Fig 3a). This finding indicates that the estimate of the juvenile abundance ratio by genotype assignment is robust to the choice of the threshold value.

## Discussion

Our genotype assignment analyses showed that larval YP from the Maumee River plume experienced significantly higher recruitment though their first summer than the Detroit River plume larvae in 2006 and 2007, but not in 2008. The recruitment advantage for the Maumee River plume larvae may be explained by at least two different processes: 1) nutrient-rich water from the Maumee River provides a food-rich (and/or high quality food) environment causing a “bottom-up” growth effect on larval fish indirectly favouring survival [6, 47]; or 2) high turbidity (i.e., low water clarity) due to suspended sediments and phytoplankton blooms in the Maumee River plume provide protection against visual predators during early life stages [48, 49]. A combination of both is also possible.

River discharge into bays, estuaries, and other coastal areas of both marine and freshwater ecosystems typically creates nutrient-rich areas that hold the potential to enhance larval growth and positively influence fish survival and recruitment [4, 14, 50]. For example, Roseman *et al*. [51], working in the southern part of western Lake Erie, showed that walleye *(Sander vitreus)* larvae were found in higher densities in waters with higher zooplankton availability, higher temperatures, and lower water clarity. Similarly, the south-shore Maumee River plume was found to have higher total phosphorous levels, chlorophyll *a* levels, and temperatures relative to other areas within the western basin of Lake Erie, including the Detroit River plume, during our study years [12, 28]. Despite these differences, zooplankton density, biomass, and production, as well as larval YP feeding, diet selection, and growth rates have been shown to be strikingly similar between the Maumee and Detroit River plumes during our study years [12, 28]. Hence, food availability that enhances larval growth does not appear to be the dominant factor driving higher recruitment of larvae to the age-0 juvenile stage in the Maumee River plume relative to the Detroit River plume.

Instead, the recruitment advantage provided by the Maumee River plume is likely due to its higher turbidity levels (relative to the Detroit River plume) that reduce predation pressure on larvae [8, 28]. The low turbidity associated with the Detroit River plume in combination with higher larval YP abundance makes opportunistic predation by piscivorous fish more likely in the Detroit versus Maumee River plume [48]. Further, lower water clarity and larval YP abundance in the Maumee River plume could translate into higher energetic costs for predators in searching for larval fish prey which also would be expected to reduce opportunistic predation [47, 52, 53]. For example, Swenson [54] reported that high turbidity associated with river discharge in the western arm of Lake Superior favoured lake herring (*Coregonus artedi*) recruitment success by protecting individuals from lake trout (*Salvelinus namaycush*) that preferred less turbid water. In the same way, higher turbidity in the Maumee River plume could be a major factor protecting larval fish from visual predators in the western basin of Lake Erie [7, 8, 28].

Another possible explanation for the apparently higher recruitment of the Maumee River plume larvae to the age-0 juvenile stage may be strong currents from the Detroit River [55] that could flush larvae and/or early juveniles from the western basin into the central basin, an area that we did not sample. Such a phenomenon would generate a downward bias in our estimates of recruitment in the Detroit River plume larvae. The effect would be magnified by Maumee River plume larvae being less susceptible to such transport, as currents generated by the Maumee River are much weaker than the Detroit River [55, 56]. However, the juvenile YP that belonged to the Detroit River plume larval group were found to be dispersed throughout the western basin, suggesting that Detroit River plume larvae were not being systematically displaced east towards the central basin. On average 17% of our larval YP were excluded from the self-assignment analysis in the Detroit and Maumee River plume larval groups. While the possibility exists that these larvae originated from some other (un-sampled) source population(s), these unassigned larvae also may have been transported by water currents away from their natal plume area into the adjacent nursery plume area before capture in our nets. Even though otolith microchemical evidence suggests that most larvae remained in the Detroit River plume or Maumee River plume for several weeks after hatching [12], some larvae (perhaps the weakest swimmers) may have been transported away from their natal areas before collection by us.

We found significant, albeit weak, spatial genetic structure among larval YP in the western basin of Lake Erie during our study years, with genetic differentiation corresponding to geographically separated larval rearing habitats (i.e., the Maumee vs. Detroit River plumes). While within-lake genetic structure has been previously reported for Lake Erie YP [30, 31, 57], these studies used juveniles and adults in their analyses, not larvae. Thus, our study is the first Great Lakes study to document that genetic structure exists during the larval stage for YP, a phenomenon that has been widely documented in other sympatric populations in marine ecosystems (e.g., [58–60]). Interestingly, Lecomte and Dodson [58] also revealed levels of genetic differentiation similar to our own (larval F_ST_ = 0.001–0.044)) between two groups of larval rainbow smelt (*Osmerus mordax*) in the St. Lawrence estuary (Canada). And, like us, Lecomte and Dodson [58] suggested that these genetic differences arose because the two sympatric groups of larvae were exploiting habitats that varied in terms of turbidity.

Our demonstration of genetic structure at small spatial scales supports previous research with Lake Erie YP [30, 31, 57], Lake Michigan YP [61], and congeneric Eurasian perch (*Perca fluviatilis*) from lakes in central Sweden [62–63]. For example, Sepulveda-Villet *et al*. [30] used mitochondrial DNA (mtDNA) haplotype analysis and found low but significant genetic divergence between adult YP spawning groups within the western basin of Lake Erie, specifically in the Sandusky Bay area versus the northern shore of the western basin. Other studies using microsatellite loci [31, 56] reported levels of genetic differentiation among spawning groups in the western basin of Lake Erie similar to the genetic divergence reported here for the juveniles assigned to the Maumee and Detroit River plume larval populations.

Additionally, we found that the level of genetic divergence between the Maumee and Detroit River plumes was greater for juveniles (juvenile F_ST_ range = 0.0143 to 0.0244) than it was for larvae in all years, with this increase in F_ST_ from larvae to YOY being insensitive to our choice of assignment threshold. The underlying mechanism for this increase in F_ST_ values between life stages is unknown; however, selection or severe bottleneck (e.g., sampling difference among life stages, as surviving adults are a fraction of the potential larvae) are two obvious possibilities. Interestingly, the juveniles exhibited reduced HWE departures (relative to the larvae) and one microsatellite locus (YP81) exhibited anomalously high divergence. This latter finding is consistent with possible linkage disequilibrium resulting from a selection differential between the two larval habitats. These results indicate that adult or spawning population genetic analysis may not reflect larval and juvenile genetic population structure or dynamics, especially in large and complex ecosystems such as the western basin of Lake Erie.

The presence of genetic structure between populations of larval YP found near the Maumee River plume versus the Detroit River plume also may be attributed to adult YP spawning site fidelity or homing [24, 31, 64] coupled with relatively weak larval swimming capabilities [42] that limits active dispersal during the first weeks post-hatching. Thus, we may be indirectly observing genetic structure among spawning adult YP. The observed year-to-year variation in the genetic structure of larvae may further reflect variation in environmental conditions at the spawning grounds and rearing sites [65], changes in cryptic barriers such as water currents [66], or differential survival or reproductive success among spawning populations [65–68]. Even though the level of larval genetic structure reported in this study is low, it indicates that genetic structure can be detected very early in life and that different life stages of fish should be included in genetic studies to better understand the relationship between habitat use and dispersal.

Otolith microchemistry is another technique that has been used to assign fish to their larval rearing habitats [69–71]. Reichert et al. [12] identified water-mass specific elemental signatures in the Maumee and Detroit River plume habitats, and these elemental signatures were used to assign juvenile YP back to their larval rearing area (i.e. Detroit or Maumee River plumes), thus enabling these authors to estimate recruitment success differences between larvae from both rearing areas for 2006 and 2007. Our genetic assignment approach agreed with the results presented in Reichert *et al*. [12] in that the Maumee River plume rearing site had a higher recruitment in 2006 and 2007, compared to the Detroit River plume larvae. While a quantitative comparison of our findings with Reichert *et al*.’s (2010) is beyond the scope of this study, both methods appear to be potentially valuable for discriminating stocks and identifying natal origins of recruited individuals.

In conclusion, we found evidence to indicate that survival of larval yellow perch through their first summer was higher for individuals residing inside a nutrient-rich, turbid river plume (i.e., the Maumee River plume) than those residing outside of it (i.e., the Detroit River plume). Our ability to document this result was provided by weak but significant genetic structure between larval YP residing in the Detroit versus Maumee River plumes coupled with temporal genotype assignment (from juvenile to larval source populations). Unfortunately, the mechanisms responsible for the larval genetic structure and the change in genetic structure from the larval to juvenile stages remain enigmatic, and thus require further study. Our results indicate that including early life stage population genetic analyses would provide a better picture of the complex interactions between nursery habitat, early life survival and genetic structure.
